# Evaluation of a family intervention programme for the treatment of overweight and obese children (Nereu Programme): a randomized clinical trial study protocol

**DOI:** 10.1186/1471-2458-13-1000

**Published:** 2013-10-23

**Authors:** Noemi Serra-Paya, Assumpta Ensenyat, Jordi Real, Iván Castro-Viñuales, Amalia Zapata, Gisela Galindo, Eduard Solé-Mir, Jordi Bosch-Muñoz, Jose Maria Mur, Concepció Teixidó

**Affiliations:** 1 National Institute for Physical Education of Catalonia (INEFC) of Lleida, University of Lleida, Partida Caparrella s/n, 25191 Lleida, Spain; 2 Unitat de Suport a la Recerca Lleida - Barcelona, Institut Universitari d’Investigació en Atenció Primària Jordi Gol (IDIAP Jordi Gol), Lleida, Spain; 3 Universitat Internacional de Catalunya, Sant Cugat, Spain; 4 Nereu’s Association, Food and Healthy Physical Exercise, Health Region of Lleida, Lleida, Spain; 5 Nursery Department, University of Lleida, Lleida, Spain; 6 Centre d'Atenció Primària Primer de Maig. Institut Català de la Salut, Lleida, Spain; 7 Department of Paediatrics Hospital Universitari Arnau de Vilanova, Lleida, Spain

**Keywords:** Obesity, Children, Physical activity, Nutrition, Behaviour, Health, Sedentary, Paediatric unit

## Abstract

**Background:**

Obesity is mainly attributed to environmental factors. In developed countries, the time spent on physical activity tasks is decreasing, whereas sedentary behaviour patterns are increasing.

The purpose of the intervention is to evaluate the effectiveness of an intensive family-based behavioural multi-component intervention (Nereu programme) and compared it to counselling intervention such as a health centre intervention programme for the management of children’s obesity.

**Methods/**Design**:**

The study design is a randomized controlled multicenter clinical trial using two types of interventions: Nereu and Counselling. The Nereu programme is an 8-month intensive family-based multi-component behavioural intervention. This programme is based on a multidisciplinary intervention consisting of 4 components: physical activity sessions for children, family theoretical and practical sessions for parents, behaviour strategy sessions involving both, parents and children, and lastly, weekend extra activities for all. Counselling is offered to the family in the form of a monthly physical health and eating habits session. Participants will be recruited according the following criteria: 6 to 12 year-old-children, referred from their paediatricians due to overweight or obesity according the International Obesity Task Force criteria and with a sedentary profile (less than 2 hours per week of physical activity), they must live in or near the municipality of Lleida (Spain) and their healthcare paediatric unit must have previously accepted to cooperate with this study. The following variables will be evaluated: a) cardiovascular risk factors (anthropometric parameters, blood test and blood pressure), b) sedentary and physical activity behaviour and dietary intake, c) psychological aspects d) health related quality of life (HRQOL), e) cost-effectiveness of the intervention in relation to HRQOL. These variables will be then be evaluated 4 times longitudinally: at baseline, at the end of the intervention (8 months later), 6 and 12 months after the intervention. We have considered necessary to recruit 100 children and divide them in 2 groups of 50 to detect the differences between the groups.

**Discussion:**

This trial will provide new evidence for the long-term effects of childhood obesity management, as well as help to know the impact of the present intervention as a health intervention tool for healthcare centres.

**Trial registration:**

ClinicalTrials.gov, NCT01878994

## Background

Obesity in children is one of the most important public health a problem in the 21st century, as it is has been voiced for years by the World Health Organization. It is considered the most common nutritional or metabolic disorder and the main non-contagious illness in developed countries. The National Health Survey in Spain [1], in its three last editions [2003, 2006, 2010], has shown a continuous increase in overweight [18.2- 18.7- 19.2%] and obesity percentages [8.5- 8.9- 9.4%], in children between 2 and 17 years old. More recently, the results of Aladinos’ study [2], indicated that 45.2% of children between 6 and 9.9 years of age are either obese or overweight.

Obesity is a complex and multifactorial cronical illness, with its origin in a behavioural and environmental interaction [3], leading to an imbalance between energy intake and expenditure [4]. It usually begins in childhood or adolescence and it is considered a risk factor for metabolic, cardiovascular and pulmonary diseases [5]. We need to take also into account the psychosocial problems of obese child [5] and their lower quality of life compared to their healthy-weight peers [6].

Due to its important health, social and psychological consequences [7], the prevention and treatment of childhood obesity has become one of the leading priorities of public health. It is critical to begin prevention during childhood as childhood obesity tends to persist into adulthood [7]; about 70% of obese children continue to be obese into their adulthood [8].

Connely et al. [9] consider that physical activity at moderate-to-high intensity is the principal factor to distinguish between effective and ineffective childhood obesity prevention programmes. However, in childhood obesity treatment programmes, performing physical activity 3 times per week was not enough to reduce adiposity [10-12]. According to Trinh [13], focusing treatment of childhood obesity only in physical activity is not enough, as its relationship to body mass index (BMI) is not clearly quantified.

Reviews from Atlantis [14], McGovern [15] and Spruijt-metz [16] have shown that 12% -14% of the programmes treating childhood obesity that include physical exercise have a positive effect on the amountof adiposity. Oude Luttikhuis et al. [17], after performing a systematic review on the interventions to treat obesity in children and youngsters, show that the most effective programmes are those which integrate different strategies in obesity management besides physical activity. They emphasize the value of family interventions involving physical activity, nutrition and behaviour. Furthermore, in a meta-analysis, Whitlock [18] adds that intervention effectiveness depends on the total length of the intervention, considering moderate (26–75 hours/intervention) to high (>75 hours/intervention) intensity interventions the most effective ones.

Thus, this increase on physical activity practice needs to be linked to changes in other important habits such as nutrition, psychological aspects and the behaviour of the nuclear family [17,19-21], the latter being even more necessary in pre-adolescent children [20,22].

These other factors can be responsible for obesity maintenance and one of the limiting factors in childhood obesity interventions.

Recent bibliographic reviews [7,14-18,21] show that this is a growing research field but there are still questions to resolve, such as the high percentage of incomplete follow-ups, which make it difficult to assess the long-term effectiveness of the programmes/interventions. In addition, paediatric units still lack effective tools to treat obesity in children. In this sense, the Nereu programme (NP) has been developed in order to give paediatric units a tool to help them in the management obesity long-term.

The aim of this study is to evaluate the effectiveness of an intensive family-based behavioural multi-component intervention (NP) compared to counselling intervention (CG; advice on physical activity and dietary healthy behaviour) as a health centre intervention tool for the management of children’s obesity.

Secondary objectives are the evaluation of the effectiveness of the intervention changes in the following parameters at short, medium and long term following the intervention referred to as baseline:

a) Cardiovascular risk factors: Anthropometric parameters (BMI, BMI SD score, waist-size index and waist circumference), blood pressure (diastolic and systolic pressure) and blood tests (LDL cholesterol, HDL cholesterol, triglycerides, glucose, insulin, TSH and cortisol).

b) Physical condition evaluated by a physical fitness test.

c) Sedentary and physical activity behaviour and dietary intake.

d) Psychological aspects such as self-efficacy and self-concept.

e) Health related quality of life (HRQOL).

f) Cost-effectiveness of the intervention in relation to HRQOL.

## Methods/Design

### Trial design

**Figure 1:**
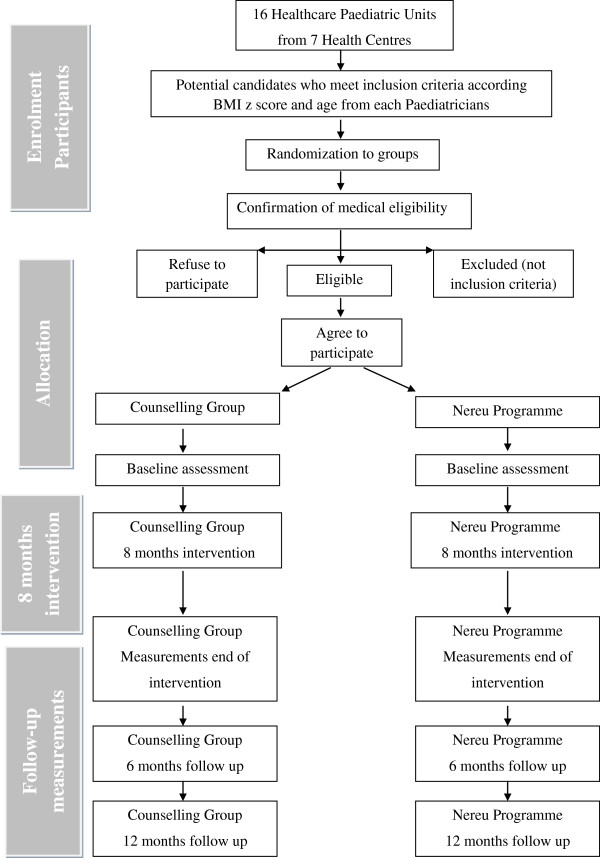
Flow design.

The study design is a randomized controlled multicenter clinical trial over a period of 20 months (Figure 1) for overweight and obese children. They will be randomly allocated to study groups previous to participant’s recruitment. The study is children and family-based and includes an 8 month intervention of physical activity sessions for children, family sessions for parents, behaviour strategy sessions for children and parents, and weekend extra activities. Briefly, at least 100 obese children will be randomly allocated to either NP or CG. An assessment will be made before the intervention, 8 months later (at the end of the intervention), and 6 and 12 months of the follow-up period. All measurements will be taken at the same research unit and by trained researchers or HPU professionals blinded to the allocated study group.

After the follow-up period, the children in the control group will be offered to participate in the next season of the NP.

### Participants

Eligible participants will be children aged between 6 and 12 years old who are overweight or obese according the International Obesity Task Force Criteria (IOTF) defined by Cole et al. [23]. They are sedentary (less than 2 hours per week of physical activity outside school hours), live in or near the municipality of Lleida (Spain) and their healthcare paediatric unit (HCP) has previously accepted to cooperate in this study. In addition, at least one of the parents or guardians of the child must accept to actively participate in the study.

Exclusion criteria are: a) medical co-morbidities, such as Cushing disease, hypotiroidism, cardiovascular diseases or other serious chronic illnesses; b) use of medication that might have an effect on weight loss or adaptations to exertion; c) previous enrolment in other obesity treatment interventions; d) regular participation in physical exercise programs in the past 6 months.

### Randomization

On a first phase, professionals of the HCP in Lleida are informed of the purpose and the methodology of the study and are invited to participate in it. Sixteen HCPs accepted to cooperate in this study. Each HPU is responsible for the recruitment of participants and for the checking of their eligibility. Randomization will be centralized at the Primary Care Research Institute (IDIAP) Jordi Gol in Lleida. Each cooperating healthcare paediatric unit (HPU) will provide a random list of their patients/children fulfilling age and BMI SD scores inclusion criteria according to the data from their health clinical records. These eligible children will be randomly assigned to one of the study groups. Randomization will insure that patients are distributed to the 2 groups homogenously in terms of age and gender. Group homogeneity with regard to age will be assured by stratified randomization according to the age group: 6,7,8,9,10,11,12 years old (7 groups) in each HPU (16 HPU).

### Recruitment strategy

Next, each HPU will phone/contact eligible families and will invite them to participate in the study. At that point participants will be informed about their study/case group. HPUs will recruit participants consecutively and on an alternate list mode basis, i.e. once they have recruited one eligible child from the intervention group list, they move on to recruit another participant from the CG list. Families of eligible children that accept to participate will be referred to their healthcare paediatric unit office for anin-depth explanation, followed by a medical assessment (basic exploration and blood test to check for inclusion and exclusion criteria). Children’s assent and written parental informed consent will be obtained from children who fulfil the inclusion criteria and with no exclusion criteria. The family will be finally included in the study.

### Sample size

The aim of the research team is to recruit 50 subjects per group, computing a total of at least 100 participants. The calculation of the sample size takes as its primary outcome intervention efficacy - the reduction of BMI SD scores after the intervention, as specified in a published meta-analytic review of trials [24]. The sample size was calculated in order to detect one BMI SD scores reduction (effect size = 0.60 [24]), according an 80.0% statistical power, 5% significance level to detect differences between groups with two independent samples. It is assumed a 20% dropout rate was estimated.

### Ethical aspects/Considerations

The study will be carried out according to the principles of the “Declaration of Helsinki” and subsequent revisions [25] and to the Guidelines for Good Practice in Primary Care Research of the IDIAP [26]. This protocol has been approved by the Clinical Research Ethics Committee (CEIC) of the Primary Care Research Institute (IDIAP) Jordi Gol. The study methods are in agreement with the CONSORT guidelines for reporting randomizated trials [27].

### Intervention

#### Nereu programme

The NP is an 8-month intensive family-based behavioural multi-component intervention (from October to May, that is, an academic year), consisting of 4 components (Table 1): (a) physical activity sessions for children, (b) family theoretical and practical sessions for parents, (c) behaviour strategy sessions, that involve both parental and child participation and (d) weekend extra activities.

**Table 1:** Contents of the assembly of children, the family theoretical counseling sessions for parents and the behavior strategies sessions for both

**Target/term**	**Parents/children**			**Parents’ sessions contents**	**Children’s assembly contents**
	**Phase**	**N**	**Behavior change strategy**		
**1st Term (October-December) Getting informed**	Concienciation (**Attention**)	1	Explaining expectations	Presenting the programme	What is Nereu Programme?
			Information on health components of the programme	Understanding the expectations of the parents at the beginning of the Nereu Programme	What do you want to know?
		2	Information about healthy food	Reflecting on the current diet and healthy food benefits	Benefits of healthy food
			Barrier identification		
		3	Information on outcomes	Making them aware of the importance of PA	Why should we take part in sport?
		4	Provide information about healthier diet	Knowing the behaviours and actions that help us improve our diet	Let's go to eat healthy and funny!
		5	Provide information about healthier behaviour	Becoming familiar with their lifestyle and how to make it more active	How can I go to the school?
		6	Explaining how the programme aims to encourage healthier lifestyles	Setting short-term goals (behaviour strategy session I)	
**2nd Term (January-March) Becoming aware**	Modelation (**Retention**)	7	Instructions about nutrition	Understanding and knowing eat quantities	The traffic light game
		8	Ways in which they can achieve a more active lifestyle/Identifying barriers to participation	Becoming familiar with their availability and requirements in order to be more PA. What we should be doing!	Family photography!! Which sport will you Take part in?
			Self-monitoring of existing PA		
		9	Provide information about healthier eating	Myths related to nutrition	What do we know about nutrition?
		10	Encouraging healthier lifestyle	Using different tools to become more active	The weather is good, let’s go and have fun!!
		11	Overcome specific barriers	Giving options to eat on specific days (Christmas, restaurants…)	Ticket-aaaa!!! Eating out side!!
		12	Social support and change	Behaviour modification strategies day-to-day	Where are we going next weekend?
		13	Provide instructions about healthier food	knowledge about how to interpret advertising and how buy food	Let’s go to buy!
		14	Provide feedback on performance	Ways in which they can incorporate PA into their lifestyle.	Why do we use the elevator?
		15	Encouragement and setting goals on PA and nutrition	Setting medium-term goals (behaviour strategy session II)	
			Maintaining behaviour strategies		
**3rd Term (April-Mai) Committing and keeping up**	Autonomy (**Reproduction**)	16	Provide knowledge about healthier eating	Learning to make a balanced menu	How do a balanced menu?
		17	Specific encouragement	Relation between PA and food intake	Burning sweets!
			Decisional balance		
	Evaluation (**Motivation**)	18	Self PA evaluation	Evaluating the implementation of PA	How active are we?
		19	Self-Diet evaluation	Evaluating the implementation of Diet	How well you eat?
		20	Encouraging PA and nutrition	Keeping up medium and long-term behaviour (behaviour strategy session III)	
			Maintenance behaviour strategies		
	Closure	21	Enjoying a healthy day together	Closing Party	
**1 each term**	3 weekend extra activities	--	Encouraging active behaviour in an experiential manner and social support	3 extra family physical activities: Ski, FC Barcelona, Aqua party	

The whole intervention will take place in 3 different school centres and health care centres, which have been recruited especially for the intervention and with a strategic localization around the city, in order to facilitate their accessibility. The children’s physical activity sessions will take place using the sport equipments of the school, the theoretical sessions for parents at the same school or at the health care centre next to the school, and the behaviour strategy sessions will be performed in both places. Parents’ and children’s sessions will be performed simultaneously in order to facilitate their attendance. All intervention groups will have a maximum of 15 children and parents.

The 4 structured components are:

a). Physical activity sessions for children

The physical exercise programme offered to children will consist of 90 sessions (3 sessions per week, each lasting 60 minutes). The main aim of the sessions is to enhance a physical active behaviour, to look for greater enjoyment during physical activity tasks and meet and practise new sports and games in order to keep practising them for a long time.

All sessions are planned to be performed in a friendly uncompetitive atmosphere and adapted to the participants’ needs, because motivating and encouraging obese children to be physically active cannot be achieved following the same approach as for normal weight children [28]. Obese children are physiologically different from those who are normal weight, and they also have significant emotional differences [29]. In that sense, the sessions have been planned by specialists with at least 4 years of experience in physical activity with overweight and obese children and following the physical activity guidelines for children [30-32]. All the sessions will be performed by two coaches who have the sport science degree with specific knowledge and experience in sport treatment for children with overweight and obesity and who have also attended the specific Nereu course. The Nereu course has been addressed specially to teach and help coaches, nurses and physical activity professionals before starting with the intervention, with specific contents about obesity management.

All physical activity sessions have a similar structure but differ in their contents. Sessions have a four-part structure: assembly, warm-up, workout and cool down periods. During the assembly, the coach explains the day’s training task, attempts to motivate children and introduces contents related to health behaviour based on behaviour change strategies. The assembly’s contents (Table 1) are the same as the family theoretical and practical sessions’ contents for parents, but taught in a playful and experimental atmosphere. Teaching the same health behaviour contents and on the same day and at the same time to both parents and children looks for an improvement in the effectiveness of these contents and their application at home by the family unit. Afterwards, during the warm-up part, dynamic activities such as walking or jogging will be performed at low intensities looking for their activation before the main part. The main part of the session (workout) is primarily focused on being physically active, but as overweight and obese children generally are not especially fit and tend both to be sedentary and tend to have had poor experiences with sport [33], exercises will be planned in short periods of duration such as 4–5 minutes of moderate-high intensity activities intersected by periods of low intensity. Short bouts of intermittent exercise are considered most appropriate for this population [34]. The sessions are also designed looking for their enjoyment through practising and learning different kinds of sports, activities and abilities. Training tasks will be mainly aerobic, but strength, joint mobility and balance will be also included (Table 2). These have been planned according to 3 essential pillars: playing, enjoying oneself and moving in order for children to get rid gradually of their fear and reluctance to sports. The cool-down period is comprised by recovery exercises and static stretching allowing participants to recover.

In addition, each session from the workout part has been planned to be a moderate-high intensity activity. In that sense, one session every two weeks in each centre will be recorded by an accelerometer and heart rate monitor and followed with an assessment in order to be sure that children from the 3 PA centres follow and reach the same indications in terms of intensity.

b). Family theoretical and practical sessions for parents

The family programme consists of 21 theoretical and practical counselling sessions with a duration of 60 minutes each. The sessions will be in group and will take place once a week atthe same time as their children’s sessions, giving the family the opportunity to exchange experiences and establish shared compromises later at home.

The sessions will be carried out by trained nurses and physical activity education professionals skilled inmultidisciplinary behaviour including physical activity, nutrition and healthy behaviours (Table 1).

The overall focus of the parental sessions is to help families to make better healthy behaviour choices mainly in terms of physical activity and nutrition inside the family unit.

c). Behaviour strategy sessions, involving children and parents

The three behaviour strategies sessions for parents and children, one each term, have been planned to reinforce the acquisition of healthier physical activity and eating habits within the family in a more experimental and practical manner (Table 1).

The contents of the family theoretical and practical sessions, the behaviour strategies sessions as well as the assembly of physical activity sessions, are planned mainly according to the Social Cognitive Theory (SCT) of Bandura [35], and the guidelines of several institutions [32,36-38].

d). Weekend extra activities

Additionally, three extra weekend family physical activities (e.g. ski or water party) will be organized, one each term following the school calendar, in order to encourage and achieve this more active behaviour in an experiential way (Table 1). Participants’ friends or relatives will be also invited to take part in the activities, looking for their social and familial support. At the same time, plans are in place to help them to achieve the minimum recommendations of 60 minutes a day of moderate-vigorous physical activity [39].

**Table 2:** Contents of physical activity training for children, family theoretical and practical sessions and behaviour change strategies

**Term**	**Children physical activity sessions**
**1st TERM (October-December) GETTING INFORMED**	Personal knowledge games
	Interaction group activities
	Collaboration games
	Traditional games
	Balance
**2nd TERM (January-March) BECOMING AWARE**	Different kinds of adapted sports without competition
	Games with alternative equipment
	Aerobic games
	Joint mobility
	Strength games
**3rd TERM (April-Mai) COMMITTING AND KEEPING UP**	Motor and physical abilities
	Aerobic tasks
	Strength exercise
	Different kinds of sports and activities
	Outdoor sports and games

#### Counselling group

Each family will be offered 8 individual monthly 10-minute-duration meetings. These sessions will take place at the paediatrician’s office and will be delivered by the child’s nurse or/and paediatrician.

The sessions’ contents will be about tips for the promotion of healthy eating and physical activity habits.

#### Measurements

As the intervention is principally focused on a family-based behavioural multi-component intervention for children’s obesity, both children and parents will be assessed. The main measure parameters are described in Table 3.

**Table 3:** Measurements

**Aim**		**Nereu and Counselling group measures**	
		**Children**	**Parents**
Cardiovascular risks factors	Anthropometry	Weight	Weight and Height
		Height	
		BMI z score	
		Waist circumference	
		Waist-size index	
		Triceps skinfold	
		Subscapular skinfold	
	Blood pressure	Diastolic and systolic pressure	NOT analysed
	Blood test	Cholesterol (LDL, HDL), triglycerides, glucose, insulin, TSH and cortisol ^1^	NOT analysed
Physical condition	Physical condition	ALPHA test set [42]	
Behaviours	Physical activity	Seven-days Accelerometry	International Physical Activity Questionnaire IPAQ [74]
		Seven days recall physical activity questionnaire (PAQ-C) [46]	
	Nutrition	24 h dietary recall (x 3 days)	Frequency consumption (CFCA –adults version)
		Frequency consumption (CFCA – children version)	
Psychological aspects	Physiological, physical and cognitive	Physical activity self-efficacy [64]	Health-specific self-efficacy [82]
		Physical self-concept (MIFA) [66]	
		Body-image: Figure Rating Scale [67]	
		Physical activity enjoyment (PACES) [69]	
Health related quality of life	HRQOL	PedsQL 4.0 [70]	
Health economic data	Cost-effectiveness	CHU 9D [73]	EQ-5D EuroQol Group [84]
Modifiers variable	Pubertal maturity	Tanner pubertal stage [88]	
	Socio-economical and demographic parameters	Some questions from de National Healthy Survey for children [89] and for parents [90]	
	Adherence	Attendance log	
	Satisfaction	Survey	

^1^the measurement will be undertaken 2 times: at baseline and at the end of the intervention.

Measurements will be assessed before and at the end of the intervention, and 6 and 12 months after the end of it.

### Children’s outcome

#### Anthropometry

Anthropometric parameters will be measured using standard practice: weight will be measured to the nearest 0.1 kg using an electronic scale (Tanita Model SECA 214, Hamburg, Germany) and height (Ht) to the nearest of 0.1 cm with a stadiometer (Seca 214, Hamburg, Germany) with children lightly dressed and barefoot. The BMI will be calculated as weight (kg) divided by squared (m^2^) height and standard deviation score (BMI SD score) will be determined from the LMS method [40]. Waist circumference (WC) will be measured in centimetres with an anthropometric tape (precision: 0.1 mm), placed horizontally at the level of the maximum abdominal protrusion at the end of a gentle expiration [30]. Waist-to-height ratio (WHtR), will be calculated as waist circumference (cm)/height (cm).

Triceps and subscapular skinfold thickness will be measured at the right side of the body with the child standing up, with a Holtain skinfold calliper (Holtain, Crymych, United Kingdom) to the nearest 0.2 mm. Triceps skinfold is a vertical fold measurement performed on the posterior midline of the upper arm, half way between the acromion and the olecranon processes. Subscapular skinfold measurents will be taken about 20 mm below the tip of the scapula diagonally (at 45º angle to the lateral side of the body). Both skinfold measurements will be performed with the arm held freely to the side of the body. Waist circumference and skinfold measurements will be done in order (not consecutively; rotating sites) and repeated three times.

#### Blood pressure

Blood pressure assessment will be performed at the level of the brachial artery of the dominant arm using an automated (i.e. oscillometric) device (Omrom) with children in a relaxed sitting position, after 3 minutes of rest. Measurements will be taken in duplicate and the last of both measurements will be recorded.

To determine hypertension, the normative values from Spanish children published by Fernández-Goula, et al. [41] will be used.

#### Blood tests

With the participants in the sitting position, blood samples will be drawn by venipuncture after an overnight fast. These samples will be used to assess cholesterol (LDL, HDL), triglycerides, glucose, insulin, TSH and cortisol levels. Blood samples will be analysed with an automated method at the laboratory of the Hospital Universitari Arnau de Vilanova, in Lleida.

#### Physical condition

To evaluate physical activity and fitness levels, children will perform the ALPHA fitness test battery [42]. The ALPHA fitness test was specially created to assess the health-related fitness status in children and adolescents within the European Union. The physical measurements of the ALPHA fitness test that will be measured from the children are: handgrip strength, standing long jump and 4 × 10 m shuttle run test. Procedures will follow the standard guidelines indicated in the test manual [42]. To measure their aerobic capacity, the 6-minute walk testwill be used [43]. This test has been validated and has shown reproducibility in obese children [43].

#### Sedentary and physical activity behaviours

Sedentary and physical activity behaviours will be assessed by means of a) the objective measurement of physical activity levels during seven days and b) the filling in of a self-report activity questionnaire.

The objective measurement of physical activity level will be done using ActiGraph GT3X + accelerometers (ActiGraph, Pensacola, EEUU). Accelerometers will be worn by participants all day for eight consecutive days; however data from the first day will be discarded for analysis. Accelerometers will be placed on a small elastic belt and positioned on the waist. Data will be collected and stored in 30-second epochs and the mean activity counts per minute will be calculated and analyzed with ActiLife 6.0 software application (ActiGraph, Pensacola, EEUU). Age and gender specific cut-off points will be used to categorize behaviours into sedentary, light, moderate and vigorous intensity activity [44]. Before its placement, a researcher will give oral and written information about the procedure to the children and family. Families will be given a contact telephone number in case of problems during the period.

Additionally, on the day of the accelerometer is removed, children will fill out the Spanish version [45] of the Physical Activity Questionnaire for Children (PAQ-C) [46]. This is a self-administered questionnaire that assesses physical activity levels in children during the last 7 days ofthe school year [46]. The PAQ-C is one of the most widely used questionnaires of physical activity level assessment and its internal consistency and validity for children has been well established [47-50]. The PAQ-C provides a summary physical activity score derived from 9 items, each scored on a 5-point scale, which is designed to collect children’s information about different physical activities and moments: (1) spare time activity, (2–8) physical education, recess, lunch, right after school, evening, weekends, and describes-you-best, (9) take the mean of all days of the week. Questionnaires will be analysed using the scoring of the PAQ manual [51].

#### Dietary behaviour

To assess and monitor the dietary status of participants, a dietary 24 h-intake-recall for three days and an eating frequency questionnaire will be performed.

The dietetic record will be done for three days, in which the families will annotate what the child has eaten during these days. It will cover two weekdays and one weekend day, and later a nutritionist will help them to interpret their annotations and power as recorded in the program by means of the quantitative dietary diary proposed by Burke, as shown by Martin-Moreno [52] revisited and updated by Willet [53,54]. Families will be individually taught how to fill out the dietary 24 h-intake form, before they carry it out at their own home.

On the other hand, children will also complete the eating frequency questionnaire CFCA [55]. The questionnaire consists of a list of nutrients or group of nutrients. Children will be asked to indicate the intake frequency (daily, weekly or monthly) of each component of the list. This may be considered as a report card on the overall quality of diet consumed. However, if the information is combined with quantitative data about mean portions, the assessment could be semi-quantitative [56-58]. This method has already been used in longitudinal nutritional studies in children [59,60] and to assess eating patterns in children [61]. Both questionnaires are included in the diet assessment survey developed by Burke and have been performed in longitudinal studies of large populations of different ages [62]. The combination of both methods has also been applied to the assessment of eating patterns in Spain [63]. Both questionnaires will be conducted by a trained/experienced interviewer.

#### Psychological aspects and physiological factors

The physical activity self-efficacy for children [64] will be used to provide a self-report of their PA self-efficacy. The scale is a specific Spanish scale that consists in 12 items and a dichotomous scale (yes or no) will be used instead of the five-point scales commonly used for this type of instruments in order to facilitate their understanding to children [64]. The Cronbach alpha consistency is .733 and test-retest reliability is .867 [64].

The physical self-concept of children will be measured with the physical self-concept scale (MIFA) by Moreno [65], as a predictor of the intention of being physically active. It is the Spanish version of the physical activity enjoyment scale [66]. It is a questionnaire composed by 5 items, especially created for children in order to know their intention to be physically active after school. The response scale is a Likert scale ranging from 1 (strongly disagree) to 5 (strongly agree).

Body image will be assessed using the Body Figure Perceptions by Collins [67]. This instrument is useful to investigate body figure perceptions and preferences among young children [67]. This measure consists in seven gender-specific line drawings of increasing size, labelled from 1 (thinnest figure) to 7 (heaviest figure). There is a specific figure for boys and girls.

Evaluation of the physical activity enjoyment will be measured according to the Spanish version [68] of the physical activity enjoyment scale (PACES) [69]. The questionnaire consists in 16 items rated from 1 (strongly disagree) to 5 (strongly agree). The PACES is for a single enjoyment factor that can be negative or positive. The results found by Moreno [68] revealed that the scale is a valid and reliable tool to measure sport enjoyment in Spanish population.

#### Health related quality of life

The HRQOL for children and parent proxy-report will be determined by the Paediatric Quality of Life Inventory (PedsQL4.0) [70]. The PedsQL 4.0 is one of the most widely used measures of HRQOL in children and adolescents aged 2 to 18 and have proven its validity in clinical and population samples [70-72]. It has 4 generic scales (Physical, Emotional, Social, School) and consists of 23 items applicable for healthy school, as well as paediatric populations.

#### Health economic data for children

The Child Health Utility 9D (CHU 9D) [73] will be filled out by all the children in order to assess the cost-utility of the intervention. The CHU 9D is a validated measure of paediatric health-related quality of life. It has been specifically developed for use with children aged 7 to 11 years and contains 9 dimensions, each with 5 levels and it is designed to be self-completed by children. The CHU 9D will be administered at baseline, at the end of the intervention (8 months later of the baseline), 6 and 12 months post intervention.

The present questionnaire will allow for aprospective economic evaluation alongside the trial with the aim of estimating the cost-effectiveness of the NP intervention versus the CG intervention.

For collecting the resource use data and on the cost linked to the NP and CG, structured observational research methods, interviews and surveys in a sample of children will be conducted.

### Parents’ outcome

#### Anthropometric parameters

Parents’ weight and height will be measured during the children assessment appointments, following the procedures previously indicated.

#### Sedentary and physical activity behaviours

Parental sedentary and physical activity behaviours will be evaluated using the short 7-day-recall self-administered Spanish version [74] of the International Physical Activity Questionnaire (IPAQ) [74,75]. The IPAQ is one of the most widely used questionnaires of physical activity level assessment and its reliability and validity for adults has been previously established [76]. The short IPAQ allows to compute a total score of the duration (in minutes) and frequency (days) of sedentary- intensity, walking, moderate-intensity and vigorous-intensity activities. Guidelines for data processing of the International Physical Activity Questionnaire [77] will be used to analyse these questionnaires.

#### Dietary intake

To assess and monitor parents’ dietary status, the adult version of the eating frequency questionnaire (CFCA) [55] will be administered. The main difference between the adult and the children version is that parentalingestion of drinks containing alcohol will also be registered [78,79].

This questionnaire has been used previously in longitudinal dietary studies and in studies relating eating patterns and biological parameters in adults [80,81].

#### Psychological aspects

To assess the nutrition and physical exercise self-efficacy in parents, the health-specific self-efficacy scales will be used [82]. The test for the nutrition and physical exercise part has been created following the same semantic structure: “I am certain that I can do xx, even if yy (barrier)” [83]. The internal consistency (Cronbach’s alpha) for the nutrition self-efficacy scale was alpha = .87 and for the exercise self-efficacy scale it was alpha = .88 [82].

#### Health economic data for parents

The EQ-5D [84] from the EuroQol Group will be filled out by all the parents to measure the cost-utility of the intervention. The EQ-5D descriptive system comprises the following 5 dimensions: mobility, self-care, usual activities, pain/discomfort and anxiety/depression. Each dimension has 5 levels: no problems, slight problems, moderate problems, severe problems and extreme problems [85]. The EQ-5D also has the EQ visual analogue scale (EQ VAS). The EQ VAS records the respondent’s self-rated health on a vertical, visual analogue scale where the endpoints are labelled ‘Best imaginable health state’ and ‘Worst imaginable health state’ [85]. The EQ-5D has demonstrated a reliability of 0.86 to 0.90 [86]. The fill out analysis of the questionnaire will follow the user’s guide [87]. As for children, the questionnaire will be administered at baseline, at the end of the intervention (8 months later of the baseline), 6 and 12 months post intervention.

Information on resource use and on the cost associated to the part of the programme for parents (economic evaluation) will be also collected using the same structured as for children.

#### Modifier variables

It has been shown that pubertal development, socio-economic and demographic parameters, the degree of satisfaction and other factors could modulate the outcome. For that reason, a part from outcome measurements, during the study, the following control information will also be recorded.

##### Pubertal stage

Pubertal stage will be assessed by the paediatrician using Tanner criteria [88] at baseline, at the end of the intervention and during the follow-up appointments.

##### Socio-economic and demographic parameters

To control the participants’ sample for these parameters, a 10 item-questionnaire has been created. Some questions have been selected from the National Healthy Survey for children [89] and for parents [90], while some other extra questions have been designed specifically for the intervention. Briefly, all these extra questions and all the questionnaires for the intervention have been satisfactorily implemented in a pilot trial, with a similar sample of children and parents during the last edition of the Nereu programme.

##### Adherence

To control adherence rate a registerof children’s and parent’s attendance to sessions will be carried out. To consider that attendance has been satisfactory each children/parent should attend at least 80% of the scheduled sessions. Those that do not attend the 80% of the sessions will be excluded of the per protocol analysis.

##### Degree of satisfaction

At the end of the intervention, children and parents will also fill out a satisfaction survey about the intervention and the coaches.

#### Economic evaluation

The economic evaluation analysis of the cost data, the CHU-9D for the child and the EQ-5D for the parent questionnaire data and the cost-effectiveness analysis will be conducted according to the current practice methods for conducting economic evaluation. The primary cost-effectiveness outcome will be the percentage of reduction in overweight and obese children. The secondary outcome will incorporate quality adjusted life years (QALYs) as an outcome measure with the primary focus being on the impact of the child and parent quality of life, measured over a time horizon of 1 year based on the CHU-9D and EQ-5Dscore collected during the trial. The results will be expressed through the incremental cost-effectiveness ratio (ICER). The ICER is a measure that compares the difference in cost and effectiveness between the two compared interventions (NP and GC). It expresses the result as cost per QALY which can then be benchmarked against established cost-effectiveness thresholds. For example in the UK, anything costing under £20,000 per QALY is deemed cost-effective. Both parametric and non-parametric bootstrap estimates of the confidence interval for the ICERs will be estimated.

### Statistical methods

#### Management and data analysis

All data will be recorded in an electronic data sheet. Two samples will be drawn; one by intention-to-treat (ITT) and another per protocol (PPT). The ITT sample will get-together all participants assigned to each group independently of whether they complete or not the entire protocol. The PPT sample will only assemble the participants who complete the entire protocol. The analysis of the results will be carried out according to the statistical analysis plan.

#### Statistical analysis plan

An initial data analysis to debug and validate the data entered in the dataset will be performed. To assess the comparability of baseline characteristics between study groups, chi-square test (or Fisher’s exact test) for nominal variables and Student’s *t* test (or the Mann–Whitney test) for continuous variables will be used.The evolution of the quantitative efficacy parameters will also be assessed, by group, for the different time periods, using a repeated measures analysis (at Baseline, 9, 15 and 21 months; see Table 1). In the case of a homogeneity violation in any relevant variable of the study groups, the multivariate analysis will be performed (Multiple regression analysis). The percentage of participants who reach to reduce their BMI SD score per study group will be evaluated, also by logistic regression models. For this analysis, the estimations will be adjusted by baseline factors such as demographic and predictive factors for the other dependent variables. As a sensitive analysis, unadjusted estimates will also be presented in different scenarios to assess the robustness of the results. The 95% confidence interval will be estimated for all parameters. Statistical analysis will be performed using the Statistical Package for Social Sciences SPSS v17 and the level of significance will be set to 5%.

#### Possible risks and burdens

This is a long term intervention that requires children and parental attendance tothe scheduled sessions as well as to the assessment appointments for the evaluation of the short, medium and long term effects. The risk of abandonment is high but we will try to reduce it by providing constant feedback to participants and their families.

On the other hand, our previous experience shows that the potential physical risks in the study are minimal. Further, the physical sessions for children have been designed and chosen especially for them. Attention has been put on enjoyment and on lack of excessively (physical and psychological) challenging situations, as well as on trying to minimize the risk of injury, even if this cannot be eliminated completely. Moreover, sessions have been scheduled to avoid overloading children. In the case that of one of these situations happens, the child will immediately be derived to their paediatrician or specialist.

## Discussion

To the authors’ knowledge, this is the first intensive family-based behavioural multi-component intervention for childhood obesity management led from the healthcare paediatrician with a longitudinal design in Spain. It targets the same principal contents for both parents and children in the easiest manner for each age group, focusing on their posterior application in their home unit. The study aims to establish a long-term healthy behaviour related to achieving a healthy weight status, physical activity and eating behaviour in order to improve their quality of life and reduce possible health problems. The study design is a randomized controlled multicenter clinical trial using two types of treatment (Nereu and Counseling) with follow-up measurements at 9, 15 and 21 months after the intervention. We believe that our study is in agreement with the principal recommendations, mentioned by Oude Luttikhuis [17], where it is recommended for family interventions to combine diet, physical activity and behavioural components focused on behavioural changes with good methodological interaction and assessing the effects in a long term period [7]. In addition, the present study has a longitudinal design with a long-term follow-up, and as it is well known, it makes it possible to gather a higher level of evidence than a cross-sectional analysis.

Following the previous editions of the Nereu program, we expect a low drop-out and the probable drop-out rate/percentage has been contemplated in the sample size. However, if we have a high drop-out and we do not achieve the sample provided, it could occur that, the outcome variables do not become statistically significant due to the lack of statistical power. However, we must take into account that paediatricians use the Healthy Child Programme which computerizes medical records of all children, and also during the recruitment period paediatricians can look for a higher sample of children if required. In the recruitment period contamination between groups could occur, as children are randomized from the same healthcare centre or district. We are going to try to prevent this by doing the NP in an external school facility and the assessment periods will be undertaken on different days.

The results from the present study will provide new scientific evidence of the long-term effects of the childhood obesity management, as well as help to know the impact of the present intervention as a health intervention tool for healthcare centres.

## Abbreviations

NP: Nereu programme; CG: Counselling group; HRQOL: Health related quality of life; IDIAP: Primary Care Research Institute – Jordi Gol of Lleida; HPU: Healthcare paediatric unit; CEIC: Clinical Research Ethics Committee; PA: Physical activity; BMI: Body Mass Index; BMI SD sore: Body Mass Index standard deviation; WC: Waist circumference; WHtR: Waist-to-height ratio.

## Competing interest

Sr. JM and IC declare that they are members of the Nereu Association. The rest of the authors declare that they have no competing interests.

## Authors' contributions

NS, AE, GG and CT designed the study. IC and AZ designed the intervention materials and study forms. IC and JM carried out all the interventions. AE, IC, NS and AZ organized and conducted all of the assessments. NS and IC supervised the implementation of the whole intervention. JR is responsible for the accuracy of the preliminary data analysis. GG, ES, JB, CT, JR, AE and NS contributed to developing the protocols and reviewing, editing, and approving the final version of the paper. All the authors read and approved the final manuscript.

## Pre-publication history

The pre-publication history for this paper can be accessed here:

http://www.biomedcentral.com/1471-2458/13/1000/prepub
